# Effects of culturally-appropriate group education for migrants with type 2 diabetes in primary healthcare: pre-test-post-test design

**DOI:** 10.1186/s12875-024-02689-7

**Published:** 2025-01-04

**Authors:** Katarina Hjelm, Emina Hadziabdic

**Affiliations:** 1 Department of Public Health and Caring Sciences, Uppsala University, P O Box 564, Uppsala, S-751 22 Sweden; 2 Department of Health and Caring Sciences, Linnaeus University, Växjö, Sweden

**Keywords:** Culturally appropriate diabetes education model, Effect of intervention, Group-based, Migrants, Observational study, Pre-post-test design, Primary care, Type 2 diabetes

## Abstract

**Background:**

The global incidence of type 2 diabetes is rapidly rising, particularly among migrants in developed countries. Migrants bear a significant burden of diabetes. However, this study is the only to evaluate the effects of a culturally appropriate diabetes intervention for these migrants on diabetes knowledge and health outcomes, adding a novel perspective to the existing literature.

The aim of the study was to evaluate the effects on diabetes knowledge, HbA1c, and self-rated health of a previously developed, culturally appropriate diabetes education model, based on individual beliefs about health and illness, underpinned by knowledge, and conducted through focus group discussions.

**Methods:**

Observational study evaluating the intervention using a pre-test-post-test design. It involved structured interviews and HbA1c measurements before, immediately after, and three months post-participation in the group-based intervention. The study included 22 migrants from the Middle East and Africa, divided into eight focus groups. The group education was conducted by a multi-professional team, led by a diabetes specialist nurse, in primary healthcare settings. Descriptive and analytical statistics applied in analysing data.

**Results:**

The findings showed that participation in the diabetes education significantly improved the knowledge levels, led to an initial change and possible short-term improvement in HbA1c (better immediate post-intervention), albeit statistically insignificant, but no change in glycaemic control over time and in self-rated health (SRH).

**Conclusions:**

The findings supported the hypothesis of improved knowledge. Moreover, the findings showed a possible initial change in glycaemic control, but no overall effect. The study showed no change in self-rated (perceived) health. Further studies involving other populations and long-term follow-ups are needed. This study highlights the importance of culturally tailored diabetes educational programmes in our multicultural society. By recognising individual beliefs about health and illness, this education programme can significantly increase knowledge and thereby contribute to improved self-care and thus, overall health. Furthermore, it is recommended for daily practice in primary healthcare, supporting healthcare professionals with a proven strategy to increase knowledge.

## Background

Type 2 diabetes has rapidly developed into what has been considered a pandemia, particularly affecting migrants (refugees and immigrants) residing in developed countries [1, 2]. This surge in Type 2 diabetes cases will lead to an increase in the utilisation of medical services for diabetes treatment, thereby having significant economic implications for the healthcare system, aside from the implications for affected individuals and their families. The most important cornerstone in self-management of type 2 diabetes is active participation in self-care, based on knowledge about the disease [1, 3, 4]. Thus, patient education should aim to enhance a patient’s knowledge and skills regarding management, empowering them to take an active role in their treatment [4–6], to achieve optimal glycaemic control to prevent complications related to diabetes [1, 3, 4]. However, presently there is a discussion about what kind of teaching method gives the best result, but few studies have evaluated different methods for teaching ethnic minority groups or migrant groups [3, 7–9]. In the UK [5] and Australia [6] a structured group-based educational programme for individuals with type 2 diabetes entitled DESMOND, The Diabetes Education and Self-Management for Ongoing and Newly Diagnosed, is used. It is one of the few initiatives that has been evaluated and shown a variety of health improvements. Some of the courses in the programme have been adapted to be used in South Asian ethnic populations, but the effect on ethnic minority groups needs to be evaluated [5]. However, neither this study nor those included in previous reviews of culturally appropriate health educations for type 2 diabetes [7, 8] are focused on migrants but on ethnic minority groups without considering their migratory background or history. Migrants are particularly vulnerable while trying to adapt to a new life-style and environment in the new country in the acculturation process [1, 2]. A previous Danish study explored the impact of a culturally sensitive diabetes self-management education and support intervention on mental and physical health of immigrants with type 2 diabetes with primary language in Urdu, Arabic and Turkish [10]. The six week programme utilizing person-centered dialogue tools showed that it effectively improved health but did not measure knowledge. Thus, this study is the only to evaluate the effects of a culturally appropriate diabetes intervention for migrants, on diabetes knowledge and health outcomes, adding a novel perspective to the existing literature.

Health education that is tailored to the cultural and religious beliefs, as well as the linguistic skills of the targeted community, and also considering literacy skills can be defined as culturally appropriate health education [7]. Research in this area has increased over the last decennium, indicating that culturally appropriate diabetes education has consistent benefits compared to conventional care with improved diabetes knowledge and glycaemic control. However, further studies to investigate successful aspects of culturally tailored education models for migrants with type 2 diabetes are needed. Additionally, new models for diabetes education should be developed and tested to determine their clinical significance [5, 7]. Despite a previously expressed need, culturally tailored diabetes education models for migrants are scarce [7, 8] or have not been evaluated [5], and their effects remain untested. Thus, the model to be tested here (see Hadziabdic et al., 2020 for further details [11] is important and aimed at filling a knowledge gap. The model differs from previous attempts [7, 8] as it focus on migrants and starts from the participants’ own beliefs about health and illness, based on their knowledge. It is conducted through focus group discussions to reach individual beliefs. Since beliefs are culturally determined and learned through socialisation [12], the model is culturally tailored and person-centred, delivered by a multi-professional team instead of having education sessions consisting of structured lectures, where the educator, usually a healthcare professional, teaches the patient about diabetes care. It also differs from previous studies as the multi-professional team also includes a physician to get a comprehensive knowledge in diabetes management. Previous research has shown that group-based education leads to improvements in patients’ knowledge about diabetes and glycaemic control [5–8, 13, 14].

Previous qualitative studies have indicated that migrants have limited knowledge about diabetes and tend to underestimate its seriousness, which negatively influences their self-care compared to Swedish-born persons [15–18]. A survey assessing diabetes knowledge confirmed this hypothesis [19]. Furthermore, individuals from non-European countries exhibited the lowest level of knowledge about diabetes. There is ongoing debate about what kind of teaching method gives the best result, but few studies have evaluated different methods for teaching migrants. Previous studies lack a theoretical base and do not consider individuals’ own beliefs about health and illness, which are influenced by their knowledge and guide their health-related behaviour [11]. Therefore, the aim of this study was to evaluate the effects on Diabetes Knowledge, HbA1c, and Self-rated health (SRH), of a previously developed culturally appropriate diabetes education model [11], based on individual beliefs about health and illness, underpinned by knowledge, and conducted through focus group discussions. Thus, the model is both individually and culturally tailored, with the aim of improving knowledge about type 2 diabetes among migrants and hereby promoting increased participation in self-care, leading to improved health outcomes. It was hypothesised that the group-based education model could change individuals’ levels of knowledge and risk awareness. This, in turn, was expected to increase their perceived self-efficacy and inclination to actively participate in self-care among foreign-born individuals diagnosed type 2 diabetes living in Sweden.

## Methods

### Design

An observational study evaluating an intervention using a pre-test-post-test design was conducted [20]. Individual structured interviews and HbA1c measurements were obtained before the intervention, at baseline, immediately after and 3 months after the group sessions. The group-based culturally appropriate diabetes education model for migrants with type 2 diabetes to be evaluated has previously been described [11].

### Sample and setting

Individuals diagnosed with type 2 diabetes who were migrants (immigrants and refugees) residing in Sweden were recruited by healthcare staff from healthcare centres in primary care (*n* = 3) located in immigrant-dense areas. Data were collected at baseline, immediately after and 3 months after the group sessions. Inclusion criteria for the study were: individuals diagnosed with type 2 diabetes (ICD E, 11; WHO [21]), aged ≥ 18 years, and with a duration of diabetes ≥ 1 year. Participants with known psychiatric diagnoses (ICD F 00- F29/F60-F 99), registered in the medical records, were excluded on the grounds that cognitive deficiency might influence the results.

Sixty-three individuals were invited, and 33 had signed up for the intervention. Of the 30 individuals (13 females, 17 males) who were identified but who did not participate, reasons for non-participation differed. One could not participate due to not being immunised, another did not attend the scheduled meeting, and a third expressed being too occupied. Two individuals resigned due to illness, four were abroad, and seven declined to participate. Four individuals did not answer the phone/could not be contacted, and the status of the remaining (eleven) was unknown (whether they could be contacted /identified).

After receiving information (either oral or written), the operation managers at the healthcare centres approved the study, and informed the diabetes specialist nurses about the study (orally or in writing). Subsequently, members of the research team participated in workplace meetings to provide additional information to the staff. The diabetes specialist nurses (DSN) then identified persons who met the inclusion criteria based on digital medical records. Invitation letters, translated into the language spoken by the individual, with information about the study were sent or given during visits to eligible participants. They were asked to fill in a response form and return it in a prepaid envelope by mail or to the staff at the healthcare centre, who then forwarded it to the researcher. The invitation letter was translated by authorised translators into the language spoken by the identified individuals. Follow-up calls, in presence of interpreter (by telephone), were made by the DSNs for reminders.

### Data collection

Data were collected from March 2015 to March 2016 and from September 2019 to October 2023, including baseline, immediately after and 3 months after the group sessions. However, the study period was impacted by two years of the Covid-19 pandemic, ranging from March 2020 to May 2022. Thus, the study was cancelled at three different time points (in the start, middle, and end of the pandemic) due to visiting restrictions in healthcare facilities, which prohibited group education sessions to be started. This was particularly relevant as individuals with diabetes were considered a high-risk group, necessitating strict measures to protect them from infection.

A registered nurse performed structured interviews (lasting about 45–60 min, including all instruments) in a secluded location at the primary healthcare centre, and in the presence of a professional authorised interpreter. Sequential interpretation techniques were applied (word-by word), with the interpreter translating what was being said literally, using the first person (I-form), remaining neutral and maintaining confidentiality [22]. During the interview, glycosylated Haemoglobin levels, HbA1c, were measured at the healthcare centre.

The structured interview guide for the whole project was developed based on previous research experiences by the research team, e.g. Hjelm et al., [15–18], literature review, and previously developed and tested instruments such as e.g. the Diabetes Knowledge Test (DKT) [23] and Self-Rated Health [24, 25]. In this study findings from the Diabetes knowledge test (DKT; see [23]), Self-Rated Health (SRH; see [24, 25]) and clinical and socio-demographic background data are reported. The interview guide was also pilot-tested, and its face and content validity were checked [20] and found to be working well.

### Intervention

This culturally appropriate diabetes education model is centred on individual beliefs about health and illness, based on knowledge, and conducted through focus group discussions comprising five sessions, held every second week, and the programme was completed within three months. These sessions were led by a diabetes specialist nurse in collaboration with a multi-professional team (diabetes specialist nurse, physician, dietician), (for details, see Hadziabdic et al., 2020 [11]. Each focus group should include 4–5 persons and last approximately 90 min, in the presence of an interpreter. A thematic interview guide is used, with broad open-ended questions and descriptions of critical situations/health problems. Participants are encouraged to discuss their individual beliefs based on their own knowledge. Healthcare staff present at the sessions answer questions, provide additional information and ensure that basic principles for diabetes care are addressed when necessary. This diabetes education model is tailored to both individual and cultural aspects and has the potential to improve knowledge about type 2 diabetes among migrants, thus increasing self-care behaviours and improving health.

The model was tested in eight focus groups comprising five education sessions, including 22 migrants (14 females, 8 males).

### Measures

The participants’ self-reported demographic characteristics included age, gender, country of birth, migration background (employment, refugee, relative), duration of residence in Sweden, whether diagnosed in Sweden or abroad, duration of diabetes, treatment received, self-reported complications related to diabetes, educational level, employment status, and marital status.

The outcome measures used for this study were HbA1c, Diabetes knowledge, and Self-rated health (SRH).

The participants’ knowledge was assessed using the Diabetes Knowledge Test (DKT), developed by the Diabetes Research and Training Center at the University of Michigan [23]. The test includes two subscales, with a total of 23 items appropriate for adults with type 2 or type 1 diabetes. In this study, only the first subscale (14 items; general part) was used, as the second subscale focuses on issues regarding insulin treatment, and only individuals diagnosed with type 2 diabetes were included. The DKT has shown good psychometric properties, with adequate validity and reliability (Cronbach’s alpha > 0.79) [23]. The questionnaire has been adapted and used, following translations, in many countries around the world [26] and among populations of different origins (e.g. [27–31]). Translation into Swedish was done in several steps to ensure preservation of the essential meaning of the items [20]. The DKT was translated into Swedish and back-translated into English by two independent professional translators. The PI for the study (first author) then reviewed the two versions and confirmed their equivalence. Interviews were performed with the assistance of professional interpreters in the respective languages.

When assessing diabetes knowledge using the DKT [23], each correct answer was awarded one point, and zero for a wrong answer or no response. The total score was calculated based on the sum of points for the general knowledge section, questions 1–14 [32].

Self-rated health (SRH) was investigated with a single question—“How do you perceive your overall health status?”—which could be answered on an ordinal five-point scale with “very good”, “good”, “fairly good”, “bad”, or “very bad”. For data analysis, responses of “very good” and “good” were summarized, as were “bad” and “very bad”. This question has been well validated and serves as a valuable summary of individuals’ perceptions of their overall health status (or SRH) [24]. The patient’s own self-rated health has been shown to predict future use of healthcare services, morbidity and mortality [24, 25, 33]. In the data analysis, responses of “very good”, “good”, and “fairly good” were summarized, as were “bad” and “very bad”.

### Statistical analyses

Demographic characteristics were reported as medians, ranges, numbers and percentages, while values were given as means (SD) [34]. Differences between measurements were analysed using paired t-test comparisons. To increase robustness against potential violations of non-normality, Wilcoxon paired tests were also carried out. The analysis of SRH was based on a dichotomous SRH measurement, indicating low or really low (responses “bad” and “very bad”) SRH vs other levels of SRH (responses “very good”, “good”, and “fairly good”). To test for differences between measurements, McNemar’s test was applied. However, readers should be aware of the limited sample size, which may affect the interpretation of this test. Statistical significance was set at *p* < 0.05. Data were analysed using the Statistical Package for the Social Sciences version 27 (SPSS Inc., Chicago, IL, USA) and R (v 3.2).

### Ethical considerations

The study was approved by the Swedish Ethical Review Authority (Dnr 2014/198–31, 2018/324–32) and was performed in accordance with the Declaration of Helsinki. Written informed consent was obtained from all participants (World Medical Association Declaration of Helsinki, 2013 [35].

## Results

### Description of sample

Participant characteristics are shown in Table 1. The intervention included 22 individuals diagnosed type 2 diabetes, comprising fourteen males and eight females, with a median age of 57 years (range 39–70 years). The majority originated from countries in the Middle East, although some originated from African countries. They had been residing in Sweden for a median duration of 11.5 years (range 3–37 years), with most being refugees and a few having immigrated due to family ties. Most were diagnosed with type 2 diabetes abroad, in their home country, receiving treatment through diet or oral agents, with a median duration of 11 years. Many of them reported complications related to diabetes affecting the eyes. Most had an educational level below primary school and were either unemployed or retired.

**Table 1:** Participant characteristics

Variable	*N* = 22
Age (yr)^a^	57 (39–70)
Gender (n)	
Male	14
Female	8
Country of birth (n)	
Syria	11
Iraq	6
Lebanon	2
Somalia	2
Sudan	1
Reason for migration (n)	
Refugee	19
Labour	0
Family ties	3
Time of residence in Sweden (yr)^a^	11.5 (3–37)
Diagnosis of diabetes in the home country/abroad (n)	13
Diagnosis of diabetes in Sweden (n)	9
Duration of diabetes (yr)^a^	11 (2–37)
Treatment (n)	
Diet	1
Oral agents	21
Insulin	0
Combination	0
Self-reported complications (n)	
Eye	12
Kidney	1
Heart	4
Lower extremity	2
Educational level (n)	
None	2
Elementary school ≤ 6 years	3
Primary school ≤ 9 years	10
Upper secondary school	4
University < 2 years	1
University ≥ 2 years	1
Employment status (n)	
Students	4
Gainfully employed	4
Unemployed	8
Retired	7
Marital status (n)	
Married	21
Widow	1
Divorced	0

^a^Values are Median (range)

For the intervention 33 people had signed up, but only 22 ended up participating. Thus, eleven individuals only participated in the baseline measurements and were subsequently interviewed. This group included seven females and four males of the same origin as in the intervention group (five from Syria, one from Palestine, three from Eritrea and two from Somalia). They were somewhat older than the participants, with a median age of 67 years (range 37–80 years), and had a longer duration of diabetes, with a median duration of 13 years (range 5–43 years). More individuals in this group were diagnosed in Sweden, and fewer reported complications related to diabetes themselves (data not shown). The reasons for not attending the intervention sessions included illness, or travelling abroad. Another reason was education sessions being cancelled, as staff in the healthcare centres expressed it was impossible to continue with the intervention due to a lack of staff and a heavy workload related to the pandemia.

Five persons (three males, two females) were lost to follow up between baseline and 3 months post-intervention due to own or relative’s illness. They did not differ in origin, age and duration of diabetes (median 57; 11 years), or self-reported complications (mainly eyes *n* = 4). There were also some loss to follow up on the different outcome variables and time points for measurement, more so in SRH, for details see Tables 2, 3, and 4.

**Table 2:** Change of the intervention; The culturally appropriate diabetes education model conducted in focus-groups, changes over time on the outcome measure HbA1C, at base-line, immediately after the intervention and 3 months post-intervention

	HbA1C							
				Paired analysis^a^ – Individual changes				
	n	Mean (SD)	Median	n	Mean individual change from Baseline (SD)	Median	95%CI	*p*-value^1^
Time point 1: Baseline	21	62.48 (17.62)	59					
Time point 2: Immediately after intervention	20	58.80 (16.31)	56	20	−4.35 (10.30)	−2.5	(−9.17; 0.47)	0.074^2^
Time point 3: 3 months after intervention	17	62.88 (25.51)	54	16	−0.56 (17.72)	2	(−10.00; 8.88)	0.90^3^

^a^Paired analysis, including only complete observations with measurements at both time points

^1^Paired t-test

^2^Corresponding Wilcoxon paired test for changes gave for HbA1c the *p*-values *p* = 0.131 and ^3^ 0.98

**Table 3:** Change of the intervention; The culturally appropriate diabetes education model conducted in focus-groups, changes over time on the outcome measure Diabetes knowledge (DKT; Diabetes Knowledge test) at base-line, immediately after the intervention and 3 months post-intervention

	DKT							
				Paired analysis^a^ – Individual changes				
	n	Mean (SD)	Median	n	Mean individual change from Baseline (SD)	Median	95%CI	*p*-value^1^
Time point 1: Baseline	22	6.82 (3.33)	8					
Time point 2: Immediately after intervention	22	9.41 (1.62)	9	22	2.59 (3.07)	2	(1.23; 3.95)	0.0007^4^
Time point 3: 3 months after intervention	14	8.21 (3.31)	9	14	1.40 (2.77)	2	(0.54; 3.74)	0.0125^5^

^a^Paired analysis, including only complete observations with measurements at both time points

^1^Paired t-test

^4^Corresponding Wilcoxon paired test for changes gave for DKT the *p*-values *p* = 0.0011 and ^5^ 0.0179

**Table 4:** Effect of the intervention; The culturally appropriate diabetes education model conducted in focus-groups, changes over time on the outcome measure Self-rated health (SRH) at base-line, immediately after the intervention and 3 months post-intervention

	Self-Rated Health (SRH)^1^			
	n	Number of Persons with Low or Really Low SRH (%)	Changes from Baseline in SRH	*p-*value^2^
Time point 1: Baseline	20	7 (35%)		
Time point 2: Immediately after intervention	18	2 (11.1%)	1 Person from Low to Low 3 Persons from Low to High 1 Person from High to Low 11 Persons from High to High *2 missing at Baseline to High* *(not included in the paired analysis)*	0.62
Time point 3: 3 months after intervention	8	2 (25%)	1 Person from Low to Low 1 Person from Low to High 1 Person from High to Low 5 Persons from High to High	1

^1^SRH: The analysis of Self-Rated Health (SRH) was based on dichotomous SRH measurement, indicating low or really low (responses “bad” and “very bad”) SRH vs other levels of SRH (responses “very good”, “good”, and “fairly good”)

^2^Mc Nemar’s test

### Evaluation of the intervention: the culturally appropriate diabetes education model conducted in focus groups

#### Changes in HbA1c

The mean value of HbA1c improved from baseline to immediately after the intervention (62.5 [SD 17.6] vs 58.8 [SD 16.3]; paired mean difference −4.35 [SD 10.3], *p* = 0.074), but this improvement did not persist at the 3-month follow-up, where it returned to a level similar to the starting point (62.9 [SD 25.5] vs 62.5 [SD 17.6]; paired mean difference −0.56 [SD 17.7], *p* = 0.9) (Table 2). Non-parametric analyses showed the same pattern. Although the results showed a possible initial change of on average −4.35 in HbA1c, this was not statistically significant and the result does not suggest a general improvement in HbA1c values over time.

#### Changes in Diabetes Knowledge (DKT)

The mean number of correct answers on the DKT showed that the level of knowledge significantly increased from baseline and post-intervention both immediately after and 3 months later (from a mean 6.8 [SD 3.3] to 9.4 [SD 1.6]; paired mean difference 2.59 [SD 3.07], *p* = 0.0007) and from 6.8 [SD 3.3] to 8.2 [3.3]; paired mean difference 1.40 [SD 2.77], *p* = 0.0125) (Table 3). Non-parametric analyses showed the same results. Thus, the knowledge improved during the intervention.

#### Changes in Self-rated Health (SRH)

The majority of participants (65%) rated their health positively (expressed as “very good”, “good”, or “fairly good”), while one-third rated their health as low or really low (summarised as “bad” and “very bad”). No significant change was found in self-rated health from baseline to post-intervention, neither immediately after the intervention nor 3 months later (*p* = 0.62 vs 0.68) (Table 4). Thus, the SRH did not change during the intervention.

## Discussion

The present study is unique as it evaluates a previously developed culturally appropriate diabetes education model for migrants [11], which is based on individual beliefs about health and illness, underpinned by knowledge, and conducted in focus group discussions integrated into daily practice in primary healthcare. The findings showed that participation in the diabetes education led to an increase in knowledge levels and resulted in an initial change in HbA1C and possible short-term improvement in HbA1c levels (better immediately post-intervention), albeit not statistically significant, but no change in glycaemic control over time and in the SRH. Thus, the findings supported the hypothesis of improved knowledge but gave no overall effect on glycaemic control and perceived (self-rated) health.

As observed in previous research, this study found that group-based education resulted in improvements in patients’ knowledge about diabetes [5, 7, 8, 14] but despite a previously expressed need, culturally tailored diabetes education models for migrants are scarce [7, 8] and have not been evaluated and their effects tested [5]. Previous intervention studies (in groups or individually) [5, 7, 8] are focused on ethnic minority groups (mainly in the USA, African-Americans), and neither distinguish migrants from ethnic minority groups nor discuss influence of migratory background, with the exception of a study on immigrants in Denmark (Urdu, Arabic, Turkish language) [10]. However, this culturally sensitive diabetes self-management education and support intervention studied the impact on health, both physically and mentally, but not on knowledge. Thus, this study fills an important knowledge gap, and only partial comparisons with previous studies are feasible.

The initial change in HbA1c, on average −4.35 although not statistically significant, observed in this study may align with findings from previous group educations interventions, albeit not culturally adapted, which have demonstrated a decline in the improvement of HbA1c over time, both in the short- and long-term [14]. It was only with ongoing education sessions or other inputs that these benefits were sustained over a longer period. This might explain why the culturally sensitive intervention for immigrants given during six weeks, and without any follow-ups [10], did not show a statistically significant improvement in HbA1c six months after the intervention. However, previous reviews on culturally appropriate health education interventions in ethnic minority groups (individually, and/or in groups) [7, 8] showed sustained improvements in glycaemic control in short- to midterm (3 months) and group educations to be more effective. The individual changes in HbA1C post-intervention (mean −4.35 [95% CI 9.17; 0.87; SD 10.3] after and −0.56, [95%CI −10.0;8.88; SD 17.72] at 3 months) was similar to results in these studies (−0.4 [95% CI −0,5; 0.2] [7] and −4.3 [95% CI −1.4; 7.0] at three months [8]), and the culturally sensitive intervention for immigrants (−1.91 [SD 4.32] after and −1.6 [SD 10.49] at three months) [10]. The level of knowledge significantly improved during the intervention and the individual changes (2.59 [95% CI 1.23; 3.95) after, 1.40 [95% CI 0.54; 3.74] at three months) even showed a better development of knowledge than in the culturally appropriate health education interventions in ethnic minority groups at three months post-intervention (0.35 [95% CI 0.10; 0.59] [8]. Given the sample size and the confidence intervals it is likely that the improvements would have been even stronger in a bigger sample. Self-rated health (SRH) remained unchanged during the intervention and previous studies have shown diverging results; neutral effects on health-related quality of life measures (albeit limited studied) in culturally appropriate interventions in ethnic minorities [7, 8] and better self-reported general health (measured vid SF12) in the culturally sensitive intervention for immigrants [10]. However, comparisons with previous studies are difficult due to clinical and study methodology heterogeneity, and loss-to follow up.

The studied education model [11] led to improved knowledge and better development of knowledge than previous culturally appropriate models for ethnic minority groups [7, 8]. With few exceptions the previous models did not use purely interactive patient-centred methods [8], many lacked a sound theoretical base, varied in length (from single session to 24 months), and were mainly delivered by lay community health workers, or by nurses, sometimes in combinations with dieticians, but no physicians involved [7, 8, 10]. Thus, this model [11] differ as it focus on migrants, is conducted through focus-group discussions to reach individual beliefs and thereby, is both individually and culturally tailored, and includes a multi-professional team also involving a physician (except a nurse, dietician, and interpreter). It has been argued that suitable education programmes should be an integral part of every treatment plan for persons with diabetes, and also include medical aspects [8]. Further, it has the characteristics found giving most sustainable results [7, 8, 14]; ongoing education sessions (every second week) in groups during three months, led by a nurse attending all sessions for continuity and developing trustful relations [7, 8]. According to the theoretical base of the model [11] health education should be executed in a learner-centred manner respecting, and being based on, cultural, social and religious values to have the greatest impact. The role of the staff is to facilitate learning by eliciting the person’s individual beliefs, stimulating interactions/discussions, and supporting with information when needed. The patient is the expert on their health, has an active role, and should be in the centre. The chosen methodology for teaching, focus-group discussions, not only facilitate learning but also support the participants by letting them share their experiences of living with diabetes and how to learn to cope with it [11]. Thus, the model includes support, shown to improve health in a culturally sensitive intervention for immigrants [10].

Although knowledge significantly improved due to the studied intervention the challenge still remains on how that improved knowledge can be translated into better physical health outcomes, and how translation of that knowledge into action can be aided. The previous culturally sensitive intervention for immigrants [10] showed that active involvement through co-creation of the target group in the development of the education, and emphasized during the implementation, lead to improved health outcomes (physically and mentally) and self-management activities of healthy diet and physical activity. The co-creation was a way to ensure that the education met the preferences, needs and resources of the group, and thereby increased the cultural sensitivity influencing health behaviour. In the present study we have developed the education model based on experiences from previous studies on individual beliefs about health and illness in different migrant groups [11], and in a forthcoming study the participants evaluations of the model will be reported (used for audit) but the feature of co-creation can be strengthened. Also auditing the data collection process can be added to reach high quality data on chosen outcomes [11]. Further, other outcome variables focused on health behaviours and diabetes self-management activities need to be considered and added for long-term follow up of the intervention. Confidence in selecting appropriate food and being able to exercise are particular elements of self-efficacy (measuring behavioural change) shown to be related to HbA1C [7, 8].

Even though the benefits of group education in terms of peer support and by sharing experiences on improved glycaemic control have been shown, persons from different communities may benefit differently from various styles of education. Group sessions, as chosen here, might be beneficial in those focused on social relationships, while not in others preferring individual sessions, e.g. due to traditions of privacy and experienced stigma of the disease [8]. Also the attitude from the healthcare provider towards the participants, whether consultative or decisive (authoritarian), need to be further studied in migrants of different origin. To have the greatest impact health education should be implemented in a manner that respects cultural, social and religious values [7, 8] why the present model proceeds from the participants’ individual beliefs about health and illness determined by cultural background [11]. Thus, it is tailored to the patients understanding/needs and aimed to develop risk awareness to influence self-care behaviour and health.

In the standards of care in diabetes [4] a systematic approach to supporting patient behaviour change efforts is recommended and whether the education model need to be complemented with additional aids in teaching, further follow-ups, and other teaching methods, as e.g. cooking classes and exercise groups, to transfer knowledge into action need to be evaluated. Finally, the introduction of the education model in the clinical area need to be given particular attention with staff being trained in changing into a person-centered approach moving from delivering information towards listening to and address individual beliefs, obstacles and motivational needs [11]. They also have to learn to moderate groups, define their own roles in the team, and that diabetes is a complex disease that need to be understood and managed in a holistic way [11]. In the focus group discussions both the influence of psychosocial factors and social determinants on health * (*the economic, political, environmental, and social conditions in which people live) should be addressed and advice adapted to leading to better physical health outcomes [4]. It is highly important to provide everyone with diabetes education being socially and culturally appropriate for their individual situation [7, 8, 11]. However, whether the team then is to be supplemented with other skills or professions, the future will tell.

The present study might have started processes of knowledge development that need to be further supported. Diabetes knowledge is a prerequisite for good self-care and can act as a mediator for behavioural change and, thus, HbA1c levels. However, knowledge achievement alone might be insufficient to promote behavioural change [4, 14]. Furthermore, when considering these results together with the initial change of HbA1C and possible short-term improvement (better immediate post-intervention), albeit statistically insignificant, and the lack of changes in perceived or self-rated health (SRH), it cannot be ruled out that there are inferences not reached due to the limited sample size affecting the study’s power. Thus, further studies involving a larger population and long-term follow-ups are needed.

### Strengths and limitations

The main strength of this study is that we evaluated a newly developed model based on a sound theoretical foundation, which proceeds from individuals’ own beliefs about health and illness, based on their knowledge, guiding their health-related behaviour (see Hadziabdic et al.,) [11]. The results contribute to the generalisability and the applicability of the model in a clinical setting within primary healthcare. A methodological limitation is that this study had no control group [20], and any causal interpretation of the changes found must be done with care. The results align with what was hypothesized but the design of the study, unfortunately has weaknesses. The original plan was to conduct a randomised controlled trial (RCT) with a Swedish control group. However, the Covid-19 pandemic heavily influenced the implementation of the study, and the post-Covid situation in primary healthcare with staff shortages and work overload further hampered the situation [36, 37]. The reality presented barriers impossible to influence why the study design had to be changed. Thus, we cannot make definitive statements about the cause of the observed changes. On the other hand, another strength of the study was the use of an observational study design and the collection of clinical data for research purposes [20].

There was a substantial attrition rate [20], with 22 of 33 persons starting the intervention after accepting taking part in the study. The reasons were related to health status, beliefs about health and illness and risk awareness of disease, as well as time constraints associated with family and job responsibilities, or staff shortages and work overload. Factors shown to be of importance for participation of culturally and linguistically diverse populations in clinical interventions [38]. Furthermore, there was some loss-to follow-up for the outcomes studied from baseline to 3 months after the intervention. Three main factors might have influenced; time, the research process, and the person investigated [20]. As the 3 months follow-up was not part of the diabetes education, the participants might not have seen the relevance to participate. The follow-up interviews were time-consuming and might have compromised time constraints associated with social responsibilities in family, work etc. and prevented the person to go for tests afterwards. Doubt about giving the correct knowledge test answers (whether right or wrong can not be determined) could have jeopardized self-perception of own diabetes knowledge level, threatening the wish to give a social desirable response (interviewer-bias), resulting in a non-response. Interviewer failed to record data giving missing values, as for SRH. Finally, a previous review identified several factors affecting diabetes self-management (e.g. attending appointments with health-care providers, glucose monitoring) among immigrants (Arabic-speaking), including beliefs (cultural, social, religious), lack of understanding and knowledge of diabetes self-management, education level, diabetes-related distress and social factors [39]. Thus, both challenges related to the individual, interpersonal dynamics, and foremost the institutional context, heavily influenced by the infrastructural context [40]. However, a strength of the study is the use of a design with an within individual analysis studying changes within individuals [20].

A strength of the study was the use of the Diabetes Knowledge Test (DKT) with good psychometric properties shown to be a valid and reliable measure for estimating patients general understanding of diabetes [23]. It has been further adapted and used, following translations [26], in populations of different origins (non European, European, Scandinavians; immigrants and not; industrialized and developing countries) around the world (e.g. [26–32]). It might be seen as a limitation that the psychometric properties of the Swedish-translated DKT was not assessed but on the other hand recommended processes were followed [20]; translation-back-translation by independent professional translators, review of a researcher/clinician (expert), pilot-test (face/content validity checked; well functioning), and interviews assisted by professional interpreters in the individuals respective language, and considered sufficient.

The sample size is restricted, but to increase robustness against distributional violations that could occur in small samples and change the findings, non-parametric methods [20, 34] were also used. Moreover, the sample included mainly individuals originating from the Middle East and some from North Africa, predominantly refugees, with a median time of residence in Sweden of 11.5 years, making them representative of the migrant population of the mid-2000s [41, 42], encompassing the two largest migrant groups.

## Conclusion

This evaluation of the developed culturally appropriate diabetes education model, conducted in focus groups, showed a significantly improved knowledge level and a possible initial change in glycaemic control but no overall effect. Moreover, there were no observed changes in self-rated health for at least 3 months post-intervention. The findings supported the hypothesis of improved knowledge but gave no overall effect on glycaemic control and did not change perceived (self-rated) health. However, due to the limited sample size and the selected study population, both with regard to attrition and loss of follow up, generalisability of the results must be done with care. Thus, further studies involving a larger population and long-term follow-ups are needed.

### Practice implications

Despite a previously expressed need, the effects of culturally tailored diabetes education models has, with few exceptions, not been evaluated in migrants. Thus, this study fills an important knowledge gap. The model, which is based on individual beliefs about health and illness, underpinned by their knowledge, and conducted in focus group discussions, is recommended for use in daily practice within primary healthcare settings. Its aim is to increase knowledge and thereby improve self-care behaviour to promote health among migrants with type 2 diabetes.

## Data Availability

Data availability statement The data that support the findings of this study are available on reasonable request from the corresponding author. The data are not publicly available due to privacy and ethical restrictions.
